# Somatic disorders in patients followed for a psychiatric disorder at the Ar Razi hospital in Morocco

**DOI:** 10.1192/j.eurpsy.2024.1726

**Published:** 2024-08-27

**Authors:** Y. BENSALAH, S. BELBACHIR, A. OUANASS

**Affiliations:** ^1^Psychiatric hospital Ar Razi, Salé, Morocco

## Abstract

**Introduction:**

Somatic disorders in patients suffering from psychiatric disorders have become an important issue in the overall care of these patients

Comorbidity studies show that 30 to 60% of patients consulted or hospitalized in psychiatry present an associated organic pathology

However, the detection of somatic conditions in psychiatric patients remains too late and this exposes them to sometimes lethal somatic complications

**Objectives:**

To evaluate the prevalence of somatic disorders in patients followed for a psychiatric disorder at Ar Razi hospital in Salé – Morocco, and to determine the associated factors

**Methods:**

We carried out a cross-sectional study with 80 patients followed for a psychiatric disorder at Ar Razi hospital in Salé presenting clinical signs in favor of an organic pathology and transferred for specialized advice to the medical-surgical services, in the period from September 1st, 2022 until August 31st, 2023.

**Results:**

Most of our patients were male (65%) with ages ranging from 18 to 65 years. Addictive behaviors were found in more than half of our patients.

The most frequent reasons for requests for advice from medical-surgical services was the suspicion of an organic cause of psychiatric symptoms in 25% of cases or the presence of an organic warning sign in 30% of cases.

The comorbidity of somatic illness and psychiatric disorder was noted in 35% of cases.

Somatic comorbidities were essentially: infections and cardiovascular diseases.

Side effects of psychotropic drugs were predominantly neurological in 40 % of cases

**Conclusions:**

Somatic comorbidities in patients hospitalized or in consultation in psychiatric hospitals are very common, often unrecognized, hence the need for early screening in order to improve care.

**Disclosure of Interest:**

None Declared

